# What it Takes to Get Passed On: Message Content, Style, and Structure as Predictors of Retransmission in the Boston Marathon Bombing Response

**DOI:** 10.1371/journal.pone.0134452

**Published:** 2015-08-21

**Authors:** Jeannette Sutton, C. Ben Gibson, Emma S. Spiro, Cedar League, Sean M. Fitzhugh, Carter T. Butts

**Affiliations:** 1 Department of Communication, University of Kentucky, Lexington, KY, United States of America; 2 Department of Sociology, University of California Irvine, Irvine, CA, United States of America; 3 Information School, University of Washington, Seattle, WA, United States of America; 4 Trauma, Health and Hazards Center, University of Colorado, Colorado Springs, CO, United States of America; 5 Department of Statistics, University of California Irvine, Irvine, CA, United States of America; 6 Department of Electrical Engineering and Computer Science, University of California Irvine, Irvine, CA, United States of America; 7 Institute for Mathematical Behavioral Sciences, University of California Irvine, Irvine, CA, United States of America; University of Vermont, UNITED STATES

## Abstract

Message retransmission is a central aspect of information diffusion. In a disaster context, the passing on of official warning messages by members of the public also serves as a behavioral indicator of message salience, suggesting that particular messages are (or are not) perceived by the public to be both noteworthy and valuable enough to share with others. This study provides the first examination of terse message retransmission of official warning messages in response to a domestic terrorist attack, the Boston Marathon Bombing in 2013. Using messages posted from public officials’ Twitter accounts that were active during the period of the Boston Marathon bombing and manhunt, we examine the features of messages that are associated with their retransmission. We focus on message content, style, and structure, as well as the networked relationships of message senders to answer the question: what characteristics of a terse message sent under conditions of imminent threat predict its retransmission among members of the public? We employ a negative binomial model to examine how message characteristics affect message retransmission. We find that, rather than any single effect dominating the process, retransmission of official Tweets during the Boston bombing response was jointly influenced by various message content, style, and sender characteristics. These findings suggest the need for more work that investigates impact of multiple factors on the allocation of attention and on message retransmission during hazard events.

## Introduction

Over the past decade, public-facing agencies and crisis communicators have shifted their formal communication strategies to accommodate new communication channels and messaging technologies. The widespread use of short messaging services on mobile devices [1] coupled with the emergence and growth of microblogging services and status updates on social networking sites [2] have resulted in new mechanisms to reach the public at risk [3, 4], broadcasting information in real time to increase public safety under conditions of imminent threat. As such, emergency messaging strategies have moved from audible sirens overhead to mobile “sirens” in the pockets of the everyday smartphone user. Little is known, however, about public receptivity to short messages under conditions of threat, nor how these messages are shared and redistributed during a crisis event.

Research on the behavioral effects resulting from short messages designed to inform the public about imminent threat and ongoing crisis has only recently begun. In their analysis of social media posts during a crisis event, Sutton et al. [5] (p. 612) introduced the concept of “terse messaging” to explain the processes that occur in environments that restrict message features as well as interactivity among message senders and receivers. The researchers define terse messages as “brief messages that are easily shared and quickly propagated, [having] the potential to reach online users in real time, disseminating information at critical points of a hazard event.” Drawing from existing empirical research on warning messages, their work has led to the development of a framework for examining the “terse communication regime,” i.e. settings in which: (1) communication takes place via short messages; (2) there is minimal opportunity for clarification of messages by the recipient; (3) there is minimal opportunity for elicitation of additional information from the sender by the recipient; and (4) there is minimal opportunity for sending of additional, follow-up messages by the sender within any given exchange. Importantly, terse regime communication has been found to occur both offline and online in emergency contexts (for examples of the former from the pre-Internet era, see e.g. [6–8]), and has distinct characteristics stemming from the constraints it imposes on information flow. Previously, Sutton et al. [9] conducted an exploratory study on short messages during a natural hazard event, identifying communication patterns occurring among the public in response to messages originated by public officials and disseminated via Twitter during a period of imminent threat. In this work they found that characteristics of short (terse) messages most strongly associated with message passing by the public did not conform in their entirety to content and style features consistent with normative guidelines (see [10]) for longer messages, such as those disseminated via broadcast channels such as television or radio. These prior studies by Sutton and colleagues set a foundation for the study of short messages redistributed under conditions of imminent threat, specifically natural hazard events. In this paper we extend the terse communication framework to the investigation of a new hazard type: terrorism.

The empirical focus of this paper is the public retransmission of terse messages that originate from official sources in response to a terrorist event. Message retransmission is a central aspect of information diffusion, with much work to date investigating its general incidence (see e.g. [11]) dependent on topic [12], sentiment [13], or receiver characteristics [14, 15]. (Throughout this paper, we will use the term “diffusion” to refer generically to the flow of information into and through a target population, “dissemination” to refer to the act of sending information to others, and “retransmission” to refer to the act of passing on messages to others that one has received from some third party. Retransmission is thus one form of dissemination, as is the posting of original messages.) Our specific emphasis in this paper is on the connection between retransmission activity and the local context of initial transmission and/or features of the messages themselves. We argue that retransmission of a given message is a clear and visible sign that the message is actively attended to by members of the public during the period of imminent threat, and hence a behavioral indicator of message salience. Message passing is also a demonstration that certain messages are perceived by the public to have some intrinsic value (being, at the very least, seen as worth sharing with others). Here we examine multiple features of messages—including their content, style, and structure—in order to identify those features that are most consistently associated with message retransmission under imminent threat conditions. We choose to focus on message retransmission rather than passive attention relationships (e.g. who Follows whom) because this provides a more direct indicator of attention to terse messages during the threat period. By examining how message retransmission varies as a function of message properties, we are able to directly examine the factors that are predictive of message amplification.

This paper provides the first examination of retransmission of terse messages from official sources in response to a domestic terrorist attack, the Boston Marathon Bombing in 2013. Using data from 31 official Twitter accounts that were actively posting during the five day period of the Boston Marathon bombing and manhunt, we examine the features of terse messages that are associated with their retransmission. We focus on message content, style, and structure, as well as the networked relationships of message senders to answer the question: *what are the characteristics of a terse message sent under conditions of imminent threat that predict their retransmission among members of the public?*

This paper is organized as follows: we begin by providing background on disaster warnings, terse communication, and the importance of message amplification via retransmission in the context of a terrorist event. Utilizing case study research methods, we then describe our research context, data collection and analysis activities. We end with a discussion of our results and suggest directions for further inquiry, connecting research findings with implications for crisis communicators.

## Background

### Warning Messages for Hazard Events

Warning messages are routinely issued by public officials in response to an imminent threat at critical time points in the hazard response process. These messages are intended to instruct the population or group at risk on necessary protective actions to make themselves safe. Warning research has largely drawn from theories of collective behavior [16] and emergent norms [17] to explain the social processes that individuals undertake following the receipt of a warning message. Warnings are interpreted and understood through social interaction and sense-making activity, which is strongly influenced by the message itself. From this foundation has grown an extensive history of research on alerts and warnings for disaster events [10] focusing on the effects of messaging channels [18], sources [19, 20], content [21, 22], and hazard type [23] on behavioral intent and behavioral actions in response to a warning message. Individuals engage in a complex process of decision making (see [19]) prior to taking protective action, that is affected by a variety of personal, social, and situational factors.

From the research record, warning scholars have concluded that the content and style of official messages are the strongest determinants motivating appropriate and timely public protective action [10]. For example, content found in effective warning messages includes the message source [19, 24], the timing at which the warning is issued and the time to complete the protective action [21, 23], the hazard type and impact on the population at risk [25], and guidance describing the protective actions that should be taken to reduce harm to life and property [22, 23]. Effective message content should also be delivered in a style that utilizes clear and specific, unambiguous language, that is accurate and consistent both internally and across messages [10]. In cases where warning messages are constrained due to the dissemination channel being used, content and style characteristics may differ; we turn to this next.

### Terse Communication

Until recently, studies of effective warning messages have focused almost entirely on relatively “long” messages (i.e. [10, 19, 26]), that is messages that are delivered over broadcast channels and are only mildly restricted in their content and character length. These warning messages, when sent via the Common Alerting Protocol, can include up to 1380 characters of text (see [27]) With the advent of social media and other short messaging channels, however, alerts and warnings have become “terse” as they have been adapted to the constraints of online and mobile device messaging.

Terse messages are brief, easily shared messages that are constrained either by medium (such as the channel by which they are relayed) or the sending context and timescale (such as periods of imminent threat when time is limited, requiring quick exchanges of information and limiting interactions). In this context, terseness is not equated with the conventional meaning of rude communicative acts, but instead is understood as quickly relayed bursts of content, necessarily constrained due to extraneous factors. Social media, such as Twitter, which is limited to 140 characters of text, is just one channel by which a terse message can be relayed. Others include SMS messages (limited to 160 characters) and Wireless Emergency Alerts (limited to 90 characters). Twitter, however, is important channel for message dissemination because it includes opportunities for networked message amplification, or retransmission, among online communicators under conditions of threat.

While terse messages distributed over Twitter during a warning period may contain some of the content elements identified as being crucial to an effective warning as described above, character limitations restrict the likelihood that all of the effective messaging elements will be included in a single message. For instance, Sutton et al. [9] examined terse messages sent in the warning period of a wildfire event to understand the impact of Twitter’s limitations on message length (and hence amount of content shared in a single message) on message style and content. They found distinct patterns in message content and style generated by official sources under conditions of imminent threat, affecting message retransmission among the public at risk. The message content and style of terse messages sent under conditions of a terrorist attack are the focus of this research.

### Terrorism Communication

The context of terrorism differs from natural hazard events due to the intent, event forewarning, and outcome of increased societal fear. Terrorist activity has been described as intentional, violent acts, targeted toward unarmed, non-combatant persons unable to defend themselves [28]. Because it is experienced by victims as “uncontrollable” [29], it results in fear response from the public who have a need for sense-making [30] and emotional support [31]. Although it is not a new phenomenon [32], recent events such as 9/11, the anthrax attacks in 2001, and the increasing visible use of improvised explosive devices (IEDs) have made public communication a top priority for crisis responders (see The 9/11 Commission Report). Internationally, IEDs are widely recognized as being a weapon of choice among those engaged in terrorist attacks; they are frequently used by terrorists throughout the world [33]. However, IEDs have been less utilized in the United States: before the 2013 Boston Marathon bombing there were relatively few high-profile instances of such attacks (e.g. the Atlanta Olympic Park Bombing in 1996 and the Oklahoma City Bombing in 1995), although there have been more numerous cases of smaller attacks aimed at abortion providers [34]. As a result, local civic leaders have had few opportunities to issue warnings or instructions about direct threats to public safety and security posed by terrorists via IEDs [35]. Even relative to other types of terrorist attacks, the hidden, latent, uncontrollable, and seemingly capricious nature of the IED threat makes it load heavily on the factors identified by [36] as particularly likely to produce fear and a subjective sense of “risk” in the public at large, creating both a demand for action and a strong affective component to event related communication.

Recent studies on public uses of Twitter in the aftermath of terrorist incidents have revealed broad and widespread public attention online [37] that may be useful for officials developing situational awareness during the event [38–40]. Our research centers on the public retransmission of terse messages originated by public officials in order to extend the theoretical framework on terse message retransmission to conditions of terrorist attack.

### Serial Retransmission of Terse Messages

Serial retransmission occurs when the recipient of a message passes this message on to another party (who may, in turn, pass the message to yet others). A major focus of early studies into rumoring behavior, serial retransmission is an important factor in the initial diffusion of information regarding disruptive events both because of its speed (competitive with broadcast media even in the pre-Internet age [41, 42] and because of its wide reach [7, 43]. For response organizations seeking to reach as many persons as possible within a target population, retransmission of formal communications is essential: extensive retransmission allows messages to reach a much wider audience than could be directly contacted, especially within a short span of time. Moreover, extensive retransmission of messages within a population increases the number of times that a given individual is exposed to each message. Such repeated exposure can increase confidence in message veracity [44, 45], which can enhance both compliance and additional message passing [46]. As a number of researchers have noted (e.g., [47–49]), exposure to messages from multiple distinct sources is often necessary to provoke both behavioral change and further message passing, making “saturation” of the target population by informally retransmitted copies or high-fidelity variants of formal communications an important goal for response organizations. To reach a broad audience with critical information during an unfolding hazard event, response organizations thus face the challenge of producing messages that not only communicate effectively to their initial recipients, but that also have a high probability of being retransmitted by those recipients to others in the target population.

The above raises the question of what features are predictive of terse message retransmission in a hazards context. Various approaches to the study of retransmission of terse messages on Twitter in a general (i.e., non-hazard) context have been attempted to date, including Bayesian techniques [50], conditional random fields [51], and classification of the properties of successfully retransmitted messages [52, 53]. Prior work in the disaster context per se has centered on sender and message content features [48, 54]. Research on terse message retransmission during periods of imminent threat (when effective communications are especially important for loss reduction) has shown that content, style, and structural factors affect retransmission rates [9, 55]. For instance, messages containing content describing the impact of the ongoing or imminent hazard, employing a well-defined hashtag used consistently throughout the event, and using imperative and instructional language received substantially more retweets, on average, than those that do not [9]. Furthermore, the features of individual accounts, most specifically, their Follower numbers, are important contributors to predicted message exposure and retransmission rates [9]. This prior work suggests a number of features that could be expected to be predictive of terse message retransmission in the terrorism case; at the same time, the terrorism context differs both in terms of protective action guidance and hazard type from these previously studied events (e.g., wildfires, storms, etc.), and it is therefore non-obvious which if any of these factors will generalize to the former setting. Recent work by [56] examines retransmission for a targeted sample of 256 tweets sent by the general public associated with specific rumors arising during the Boston bombing, finding positive effects on retransmission for the Follower counts of the most influential posters and for hashtag usage. This work suggests that Follower count and tweet structure effects may generalize to the terrorism case (at least for messages originating within the general public), but leaves open the role of other factors—and of whether those effects continue to operate in the same manner for messages disseminated by official entities. The remainder of this paper thus seeks to address the following basic question: what are the content, style, and structural characteristics of terse messages, disseminated by emergency management organizations under conditions of imminent threat, that predict their retransmission among the public during a terrorist event?

## Methods

Utilizing a case study approach [57], we investigate the dynamics of terse message retransmission over a defined period of time for a specific event. The case study approach is suitable for answering research questions such as *why* and *how* things are done [58]. Case studies are also important as building blocks for subsequent meta-analytic studies. We are primarily interested in answering questions related to salient features of formal warning messages, including their content, style, and structural features, and how this affects message retransmission by the public. The primary data used for this study are publicly available messages posted to Twitter by official response agencies during the period of threat of the Boston Marathon bombing and manhunt. To address our research questions, we conduct thematic content analysis and statistical analyses on the set of 698 messages produced by 31 official accounts over the five day period and model their predicted retransmission rates by members of the public. In this section we provide a description of the terrorist event and public safety response, then turn to a detailed discussion of our online data collection methods, data coding, and analysis.

### The Boston Marathon Bombing and Manhunt

On Monday morning, April 15, 2013 two improvised explosive devices were detonated near the finish line of the Boston Marathon, killing three spectators and injuring more than 200 people. During the week that followed, memorials were held (April 17, 2013), two suspects were identified (April 18, 2013), and a shootout occurred resulting in the death of the first suspect as well as a MIT campus police officer (April 18, 2013). The events lead to shelter in place orders and more than a million people across the city of Boston and contiguous areas were placed on lockdown for close to 24 hours while a massive search was undertaken. The manhunt resulted in the capture of the second bombing suspect in the late hours of the day on Friday, April 19, 2013.

Over the course of the week, social media sites, Twitter in particular, gained significant attention due to their utilization by both the general public and government officials. Popular media sites posted articles about social media use by local public officials [59]. The reports noted the benefits and drawbacks of such community engagement; members of the public tweeted police chatter [60] and banded together in attempts to identify suspects from images captured at the scene of the event [61]. In addition to popular media accounts of the heightened use of Twitter, previous research also supports this convergence of attention online [62]. Key Twitter accounts held by local public officials experienced dramatic surges in attention as they provided real time updates from the scene of the bombing and additional messages throughout the week. Boston Police gained 273,000 new Followers, Massachusetts State Police picked up nearly 26,000 Followers and the Mayor of Boston, Tom Menino, experienced an increase of nearly 17,000 Followers [55].

### Data Collection

In this research, terse message retransmission is analyzed by investigating aspects of messages posted via the microblogging service, Twitter. Twitter represents one online venue for social interaction and information exchange in disasters for members of the public and public officials alike. Twitter is a social media platform that enables individuals to post terse (140 character) messages in real time. The platform facilitates information exchange through a set of publish/subscribe relationships, called *Following* relations. Public content on the platform is searchable allowing users to seek out information and other users of interest. Moreover, the platform itself enables retransmission of content with a single action; *retweets,* as they are called, allow users to rapidly retransmit information to the public sphere as well as their own personal network in real-time. These features make Twitter an ideal data source through which to examine terse messaging and message amplification in social networks.

Our data collection processes replicate those used previously [62] in order to develop a cross-hazard comparison between cases. For this event, we identified 31 Twitter accounts representing the population of public officials at the local, state, and Federal level who were serving in a public safety capacity prior to the marathon and actively tweeted over the course of the five day period. The set of accounts satisfying these criteria were identified through two processes. First, we searched through our set of user accounts that were already in our data collection system and were within the geographical boundaries of the Boston region, the state of Massachusetts, or represented Federal agencies having a role in terrorism and disaster response. Secondly, we manually sifted through the Twitter “Friend” lists of local official organizations (i.e., organizations that are Followed by the accounts we selected) to identify additional accounts that may not routinely tweet, but could play a role in relaying public information, and we looked for any account that was mentioned or retweeted in posted content from the official accounts. We did not choose to include local media as part of our targeted accounts because our interest lay in the messages posted by public officials, as part of their formal communication strategy during a terrorist incident. In total the set of targeted accounts represents 17 local public officials or organizations, 10 state actors, and four federal entities.

For each account, we retrieve the posting behavior history, along with actor level attributes, using the Twitter API. Twitter’s API allows us to obtain up to 3,200 of the most recent messages posted by the user of interest to the public timeline and the timestamp for each post. Data was queried daily over the five day period of the unfolding event to ensure no messages were missed, resulting in a complete dataset of official messages posted to Twitter. We removed any retweets from this set as they are not original content produced by these users. For each message we also obtained a count of the number of times that each message was retweeted (by the time of last data collection). Actor or user-level attributes collected include the number of Friends and Followers of that account at the time of collection, the self-reported location of the user, the account creation date, the timezone of the account, and the number of statuses posted over the lifetime of the account. For the subsequent analysis, we consider the set of 698 messages posted by our targeted accounts, from April 15, 2013 2:49pm (the day and time of the bombing) until 11:59pm on April 19, 2013 (after the manhunt was concluded).

### Content Coding

Data analysis in this work centers on an examination of the features of terse messages disseminated by officials over the course of the five day period of threat, beginning on the day of the bombing and throughout the manhunt, concluding when the suspects were captured. We conduct a thematic content coding, based upon effective message content and style elements described above, to identify variables that may predict message amplification via public retransmission. Variables include content themes, message style, and network features of posted accounts.

Coding strategies for primary thematic content analysis and message style characteristics replicate those previously conducted by Sutton et al. [62], for cross-hazard comparative purposes. In this case, two researchers manually coded the entire set of official tweets for the observation period, utilizing a deductive content coding strategy that drew from codes that were developed during previous research activities on terse messaging via Twitter during a wildfire event [62]. Both coders were blinded to the retweet count information before and during the coding process, and content codes were hence determined independently of the outcome of interest. To begin, the coders independently scanned all tweets to determine that the original coding categories fit with the Boston event data. They also met to discuss any emerging themes. Next, the set of tweets was split-recoded by both coders, with one half being blind recoded by each researcher and then exchanged and checked for intercoder agreement. Coders agreed on theme codes in approximately 98% of cases. Disagreements were resolved by consensus, following discussion of problematic cases by the coders. Coders ultimately identified 10 primary themes (plus two additional categories; one for tweets that were not on-topic, i.e. pertaining to the Boston event, and one for tweets that did not fit into any category). Primary themes range from evacuation guidance and sheltering in place to hazard information (such as listings of phone numbers and resources). A full list of content themes can be found in Table 1.

**Table 1:** Content analysis coding categories for messages from Boston Marathon Bombing.

Thematic Content	Definition and Example Tweet
Advisory	Messages containing advisory information such as requesting that people clear the area of the bombing and the actions to take while the city was on lockdown
	*#MediaAlert: WARNING: Do Not Compromise Officer Safety by Broadcasting Tactical Positions of Homes Being Searched*
Closures/Openings	Messages containing information on closures/openings of events, facilities, or roads.
	*ATTENTION: The #MBTA is SUSPENDED on ALL modes until FURTHER NOTICE*
Corrections	Messages containing corrections to previously posted information
	*We now know @bstonmarathons is a scam. We should know better and apologize for perpetuating the exploitation of the bombing. @embarrassed.*
Evacuation/shelter in place	Messages that provide specific guidance about how to protect oneself in disaster, in this case, sheltering in place
	*ATTENTION: City-wide shelter in place advised. Armed and dangerous person(s) still at large. Police actively pursuing every lead.*
Hazard Impact	Messages containing descriptions of the hazard itself such as location, containment, etc., and descriptions of the hazard impact such as number of injuries.
	*176 people when to area hospitals #tweetfromthebeat*
Information	Messages containing updates, available resources, and images of the suspects
	*@healthyboston has some tips for helping children cope with yesterday’s events http://t.co/TgykZFNYSS #bostonmrathon*
Help/Directed Communication	Messages directly responding to a member of the public requests for assistance or information.
	*@Kend129 1–93 is open*
Thank You/ Appreciation	Messages that include statements of thanks and appreciation
	*@RedCross: Thanks to generosity of volunteer blood donors there is currently enough blood on the shelves to meet demand. #BostonMarathon*
Volunteer/donate/help	Messages that suggests ways to volunteer or donate to the disaster response efforts
	*AG’s Office Offers Tips to Giving Wisely after Marathon Tragedy http://t.co.bC96Ysl4Ex @bostonmarathon #mapoli*
Emotion/Judgment/Evaluative	Messages containing emotive statements about the event, the response, and the recovery efforts
	*Yesterday was a very sad day for our city. Thank you to all first responders & spectators for quick thinking & heroic acts. #bostonmarathon*
Unsure/Not on topic	Messages that are not directly related to the Boston Marathon Event response, or could not be determined to be related
	*@tedslater we have asked facilities to check the HVAC thanks for letting us know.*

Following methods used in previous research in this area [62], two researchers also manually coded each tweet for aspects of message style. Style aspects, which emphasize how content is relayed or displayed to affect message specificity or clarity [10] include the following: (1) how each sentence in the tweet functions within the English language as either declarative, imperative, interrogative, or exclamatory; and (2) whether a tweet includes a word or phrase in ALL CAPS we distinguish between capitalizations used as either a category signifier or to emphasize a portion of the tweet.

In addition, we used automated techniques to code for conversational microstructure elements within the tweet (i.e. conventional aspects of Twitter-based syntax that lend to message retransmission or engagement) [62]. These include whether the tweet was directed at or responding to another Twitter user (begins with @name), contained a mention of another user, contained a hashtag keyword, and referenced further information available online in the form of links to URLs (usually shortened by using bit.ly or another short URL service).

For both thematic content and style features, messages were coded in a non-mutually exclusive manner; in other words, a single tweet could contain several types of content as well as multiple sentence features or other stylistic aspects.

### Measuring and Modeling Message Retransmission

A central observation of our and prior work (as cited above) is that not all messages are equally likely to be passed on by others; we thus seek to identify the factors that enhance or inhibit message transmission, by means of statistical analyses. Our analyses are in turn based on a basic model of the retransmission process, which may be summarized as follows. Consider an original message, broadcast from a targeted account to the public stream. The message has particular style and content features (as described above), each of which may serve to enhance or suppress the probability that a given message recipient will pass it onward. Likewise, the probability that a recipient will retransmit the message may be positively or negatively affected by characteristics of the sender (e.g., the type or prominence of the associated organization) and/or by the context in which the message was sent (e.g., the number of individuals following the sending organization at the time of transmission, or whether the message was simultaneously posted by multiple organizations as part of a deliberate amplification strategy). Finally, there may be additional, idiosyncratic factors relating to unmeasured and/or unpredictable aspects of the communication setting that also impact retransmission probability. In the context of this study, we note that the number of persons at least peripherally exposed to any given message is generally quite large, and that the probability of message passing by any given individual is generally quite small; given any fixed retransmission probability, we thus expect the number of times a given message is passed on (the *retweet count*) to be approximately Poisson distributed. Note, however, that the presence of idiosyncratic (i.e., random) factors implies that the retransmission probability for a message with the same observable characteristics will fluctuate from one occasion to another; a natural model for this variation is the gamma distribution, leading to a final retweet count distribution which is negative binomial given the observed message, sender, and contextual features.

Under the above model, the effects of message, sender, and contextual features on the expected retweet count can be estimated by negative binomial regression. As an additional test on the assumptions underlying the above process model, we also compared our results to regression models based on Poisson and geometric distributions. The former model corresponds to a process like the above, but without idiosyncratic variation in retweet probability; the latter model corresponds to a sequential process in which messages are passed serially with some given probability from one user to another, until the “passing chain” fails (at which point no further retransmission occurs). Neither the Poisson nor the geometric model were favored over the negative binomial model using the corrected Akaike Information Criterion (AICc), a standard model selection index. The negative binomial model, with an AICc of 7876, had a substantially lower score than the Poisson model (817655) and the geometric model (8027). In addition, we favored the negative binomial model specification over Poisson due to overdispersion of the dependent variable. We tested for this using Cameron and Trivedi’s Test for Overdispersion [63], the null hypothesis being that the variance of the dependent variable is equal to the mean. The z-score for this test was 5.434 with a p-value < 1*e*−7, suggesting that a Poisson model (which assumes a mean equal to the variance) was not appropriate. This suggests that neither alternative process provides a better account of the observed data. Finally, inspection of the data also indicated that most retransmission occurred as a single step, rather than via long chains of sequential message passing, in line with our above theoretical model. We thus note that our choice of analytic procedure is not merely one of convenience, but is founded on a specific model of the communication process that was found to outperform theoretically plausible alternatives.

Given the above, our analysis proceeds by modeling the log of the expected number of retweets for each original message as a linear function of message, and context covariates (as described below). Because sender effects (i.e., differential propensities for messages to be retransmitted as a function of sender) can come from many strongly correlated attributes (e.g., total number of statuses, local/state/federal status, government sector, number of reciprocated ties “Friends,” perceived prominence and reliability, etc.), not all of which can be measured, we include fixed effects for each sender as additional terms in the model; this controls for sender-level heterogeneity. Coefficients representing the strength of each effect are then estimated by negative binomial regression, with best-fitting models selected by AICc.

## Results

### Modeling Message Retransmission

As discussed in the methods section above, we built a model of message retransmission to assess the relative influence of content and style elements, as well as message exposure, on the number of times a message is retweeted among the public. We use the R statistical computing platform [64] to fit a negative binomial regression model for these data. As noted above, the negative binomial family allows us to account for observed overdispersion in the retweet rates relative to either a Poisson or geometric family, and is consistent with a process in which there are many sources of heterogeneity in the retweet process (only some of which can be captured via observed covariates).

Table 2 shows the result of the model selection process. Each of the primary content theme codes, stylistic features such as the use of capitalization or sentence type, structural elements such as directed messages and links, and account characteristics (e.g the number of Followers of the account posting the message) are considered as potential predictors in our model. In the table below we show the top model based on the small-sample-size adjusted Akaike Information Criterion (AICc), a model selection index that considers both goodness-of-fit to the observed data and model parsimony (in particular, the risk of overfitting). This criterion is minimized for the best fit model (i.e., lower AICc values indicate models that fit better given the number of parameters they employ). We note that inclusion of additional model terms did not result in qualitatively different results.

**Table 2:** GLM negative binomial model using source, style and theme variables predicting number of per-tweet retweets during the Boston Marathon Bombing.

		Estimate	exp(*β*)	Std. Error	z value	Pr(>∣z∣)
	(Intercept)	-18.18***	0.00	2.63	-6.91	0.00
Source						
	Source Fixed Effects^*t*^ log(Followers)	2.50***	12.21	0.30	8.33	0.00
Tweet Style						
	Directed Tweet	-2.42***	0.09	0.22	-10.79	0.00
	Flagged Third Party	-0.60***	0.55	0.15	-3.97	0.00
	Incl. URL	-0.44***	0.64	0.12	-3.61	0.00
Theme						
	Advisory	0.70***	2.02	0.15	4.78	0.00
	Closures/Openings	-0.53***	0.59	0.18	-3.02	0.00
	Evacuation/Shelter	-0.50*	0.60	0.23	-2.23	0.03
	Hazard Impact	1.17***	3.21	0.27	4.36	0.00
	Thank You	-0.75***	0.47	0.23	-3.29	0.00
	Emotion/Evaluative & Evaluative	1.29***	3.62	0.20	6.40	0.00
Use of ALL CAPS						
	EMPHASIS	0.42	1.52	0.23	1.82	0.07
	SIGNIFIER	0.61*	1.85	0.25	2.48	0.01

^*t*^ *Note:* Although not shown here, source accounts (excluding ‘Alert Boston’ for a baseline) are included as dummy variables to directly estimate fixed effects. Table 3 below shows these effects.

Dispersion parameter: 2.07 (Theta = .56)

Null Deviance: 9398 on 697 degrees of freedom.

Residual Deviance: 7802 on 664 degrees of freedom.

AICc: 7876

* *p* <.05,

*** *p* <.001

For the top model, we show the regression coefficient estimates for each variable in Table 2, along with the standard error estimate, *z*-score, and *p*-value. The residual deviance of the model is 7802 on 664 degrees of freedom, a substantial improvement over the null deviance of 9398 on 697 degrees of freedom. Included variables were also cross-checked with repeated applications of the model selection process while holding out a random subset (10%) of the data; the final variables in the reported model were included in the final models in the replicated data sets at least 95% of the time (out of 1000 replications), suggesting that the results of the AICc selection process are fairly robust. Each of the content elements included in the model has been discussed in detail in previous sections. We also include the logged number of incoming Followers of the sending account at the time each original message was posted; the Follower count is an aspect of network structure that we predict to be associated with increasing message exposure, and hence increased retweet rates. As shown in Table 2, incoming ties do indeed have a positive effect on the number of retweets per message (with a doubling in the number of Followers increasing the expected number of retweets by a factor of approximately 5.66). As noted above, we account for unobserved heterogeneity between source accounts that may affect the dependent variable via sender-level fixed effects. The reference organization here is the ‘AlertBoston’ account. (One account, ‘NWSBoston,’ showed too little posting activity during the period for its conditional mean to be reliably estimated, as reflected in the large standard error for its fixed effect within Table 3. We retain it here for completeness.) The negative binomial coefficients are interpreted as affecting the expected log count of the number of retweets. For example, a message containing emotion, judgment, or evaluative content increases the expected log count of the number of retweets by 1.29, i.e. increasing the expected retweet rate by 2.62 times compared to a tweet that does not contain emotion, judgment, or evaluative content (all else held constant). To aid in interpretation of these effects (especially in the context of multiple predictors), we find it helpful to consider the predicted retweet count for various predictors interest, reported in percentages. To simplify interpretation, we describe effect sizes here in terms of the number of additional retweets that would be gained or lost relative to the baseline upon adding or removing a message feature. Thus, a feature that multiplies the expected retweet rate by a factor of 1.5 is described as adding 50% more retweets, while a feature that multiplies the rate by a factor of 0.75 is described as resulting in 25% fewer retweets. Effect sizes stated in terms of multipliers may be found in Table 2. We discuss some of these variables presently as they correspond to the primary question: what makes a difference in the behavioral outcome of retweeting; message thematic content, style features, or network exposure (Follower count)?

**Table 3:** GLM negative binomial fixed effects predicting number of per-tweet retweets during the Boston Marathon Bombing.

	Estimate	exp(*β*)	Std. Error	z value	Pr(>∣z∣)
(Intercept)	-18.18***	0.00	2.63	-6.91	0.00
BOSTON_EMS	-0.61	0.54	0.35	-1.77	0.08
BostonFire	-3.07***	0.05	0.69	-4.42	0.00
BostonLogan	-0.72	0.49	0.58	-1.24	0.21
BostonParksDept	1.17	3.24	0.68	1.74	0.08
Boston_Police	-4.64***	0.01	1.04	-4.45	0.00
CherylFiandaca	-0.18	0.83	0.38	-0.49	0.63
DHSgov	-9.10***	0.00	1.93	-4.72	0.00
FBIPressOffice	-8.87***	0.00	1.46	-6.09	0.00
fema	-8.30***	0.00	1.74	-4.78	0.00
femaregion1	-2.51*	0.08	1.05	-2.39	0.02
HealthyBoston	-2.43***	0.09	0.65	-3.72	0.00
MassAGO	-1.49	0.23	1.39	-1.07	0.29
MassDOT	-2.20***	0.11	0.59	-3.74	0.00
MassEMA	-1.18*	0.31	0.52	-2.29	0.02
MassGovernor	-4.17***	0.02	0.77	-5.42	0.00
MassGuard	0.03	1.03	0.74	0.04	0.97
MassStatePolice	-2.80***	0.06	0.67	-4.18	0.00
mayortommenino	-1.77***	0.17	0.63	-2.83	0.00
mbtaGM	-3.02***	0.05	0.64	-4.73	0.00
MDARCommish	2.02	7.56	1.76	1.15	0.25
NotifyBoston	-2.21***	0.11	0.57	-3.87	0.00

Dispersion parameter: 2.07 (Theta = .56)

Null Deviance: 9398 on 697 degrees of freedom.

Residual Deviance: 7802 on 664 degrees of freedom.

AICc: 7876

* *p* <.05,

*** *p* <.001

**Fig 1:**
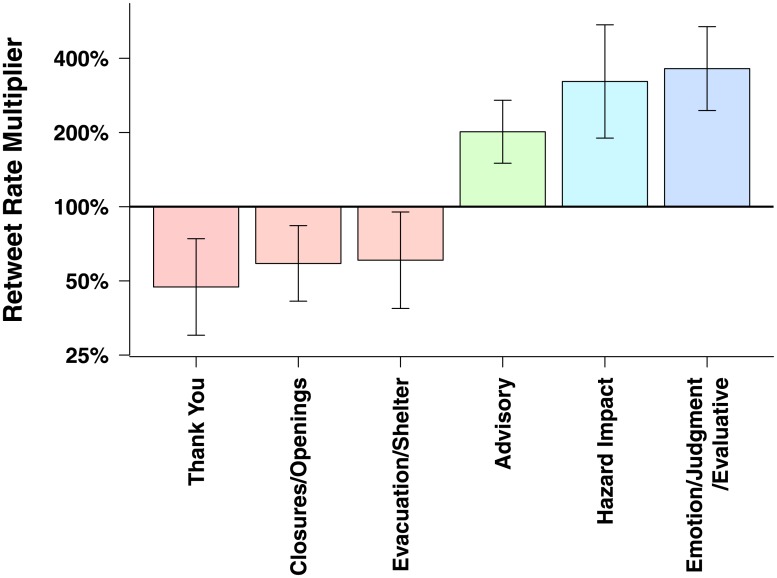
Thematic content of official messages is strongly related to expected retweet rates. Solid bars indicate the estimated retweet rate multiplier associated with the presence of each content type; error bars depict 95% confidence intervals. Tweets containing content related to advisories, hazard impact, or emotional, judgmental, or evaluative statements were on average retweeted at 2–3.5 times the rate of messages without such content. “Thank you” messages, by contrast, were retweeted at just under 50% the base rate.

First, we address the extent to which thematic message content affects the predicted number of retweets in our observed data. These effects are summarized graphically in Fig 1. We find that messages containing *hazard impact*, *advisory*, or *emotive/evaluative* thematic content are the strongest predictors of message retransmission. Messages that contain content on hazard impact are predicted to result in, on average, 221% more (i.e., additional) retweets than those tweets not on that topic (all else held constant). Those that contain advisory information, instructing people on what actions to take, are predicted to result in approximately 102% more retweets. Those that contain emotive/evaluative content, including tweets that provided encouragement or restored confidence, result in 262% more predicted retweets. By contrast, messages containing content on closures or openings, including transportation system information, are predicted to have about 41% fewer retweets, all else held constant. In addition, Tweets that include content about *thanks and gratitude* are predicted to have 53% *fewer* retweets than others.

We next consider whether message style or the inclusion of conversational microstructure elements affects the predicted count of retweets in our models. We find that the inclusion of all three microstructure elements, directed messages, a flagged third party, and the inclusion of a link, had a negative effect on predicted retweets. In our model, directed tweets (messages directed to a single individual) have 91% fewer predicted retweets than those that are not directed; tweets with a link, URL, have 36% fewer predicted retweets than those without; tweets that flag a third party are likely to have 45% fewer predicted retweets than others. Tweets using ALL CAPS as a signifier have 85% more predicted retweets as those without; those using ALL CAPS to emphasize a portion of the message, however, were not predicted to have significantly more retweets than those without such emphasis.

Finally, we also address the effect of network structure (specifically, the number of Followers) on predicted retweets. The number of Followers associated with sending accounts at the time of message broadcast is significantly related to the number of retweets (even net of other sender-level characteristics). Messages disseminated from accounts with more Followers have more predicted retweets, all else held constant. For every logged unit increase in the number of Followers, the number of predicted retweets from each tweet increases by 1,121% (Put more intuitively, doubling the Follower count increases the expected number of retweets by a factor of about 5.67.) This is an extremely powerful effect: organizations with many Followers can garner much more attention—and retweets—than those with few Followers. It is also important to bear in mind that this effect implies much more room for change in attention by small and/or local organizations than large, highly visible national ones. This is because proportionately large changes in Follower count are much easier for organizations with few Followers than those with many (an effect also observed by [62] in other hazard events). Low-visibility organizations that increase their Follower counts by a large factor over the course of an unfolding event are predicted to see a dramatic explosion in retweet rates, while established players (e.g. the Federal Emergency Management Agency, FEMA) with many Followers will typically see little change. This has important implications for Public Information Officers or other authorities within low-visibility organizations, who may encounter a sudden shift in attention to their posts that vastly exceeds past experience. We turn to these implications in the section that follows.

## Discussion

This research focuses on message retransmission as an indicator of public attention in response to official messages sent during a period of imminent threat. Building on prior work that laid out a research framework for analyzing “terse messages,” our analysis centered on the official messages communicated via Twitter in response to a terrorist incident and the five day threat period that continued post bombing event. Our research has shown that terse messages were disseminated and amplified via Twitter in the immediate aftermath and throughout the investigation and manhunt following the Boston Marathon bombing. From these we have identified a set of message features that affect predicted message retransmission among the public, contributing knowledge to the terse communication framework and providing valuable lessons about effective message design for terse communication channels in response to an terrorist event.

The messages generated and disseminated by public officials during the threat period spanned a variety of content areas, most of which were consistent with findings from other recent non-terrorist events [55, 62]. Importantly, two of the three thematic content areas that held salience among the public, as represented by predicted message retransmission, appear to hold consistently regardless of hazard type. Terse messages that include information about hazard impact and public safety advisories hold high importance among the public attending to social media messages under a period of imminent threat. One additional thematic area, messages with “emotive” type of content (content that could also be interpreted as encouraging or resiliency-building) was also found to predict retransmission rates. This differs from prior research in suggesting that a number of factors that may contribute to message salience, including the type of hazardous threat and the geographical scale of the audience attending to messages online in a disaster event.

Terrorist events often occur suddenly and without warning; they expose many to horror, appear to be beyond the control of any one person, threaten life and the lives of family members and friends, and place excessive demands on coping [65]. Research suggests that many of these characteristics are associated with survivors’ reporting that they feel some loss of their sense of control, predictability, safety, and trust [66, 67]. Because of the potential for negative psychological effects, public-facing communicators are often advised to disseminate messages that promote social cohesion and resilience [68]. In the aftermath of the bombing and throughout the manhunt, public officials used Twitter as one channel to assure the public that government agencies were responding collaboratively and expeditiously while exhorting the community of Boston that they are “one Boston” and “Boston strong.” Many of these restorative messages also demonstrated empathy, acknowledging sadness and respect for the victims of the bombing, while remaining vigilant about the pursuit of the perpetrators. It is possible that the emotive messages disseminated from official accounts served as a type of supportive intervention among the public, something that held high value among observers both near and far.

In contrast, messages that included words of gratitude or thanks were predicted to have lower rates of retweeting. Many of these messages were directed to individual persons or organizations, referencing specific situations in comparison with the emotive messages that were directed to a broader audience and, apparently, designed to uplift the entire community that was simultaneously recovering from the initial attack while remaining vigilant of further terrorist activity.

Messages dedicated to disseminating practical warnings on closures/openings, as well as tips on evacuation/shelter, had lower rates of retweeting. Most of these tweets were posted during the widely publicized manhunt, which was covered in other media forms. Since most people in the region were already locked in their homes, there would be little need to retweet this kind of information. The closures/openings reflect something similar to the evacuation/shelter in place tweets. During the manhunt, everything was shut down, with information distributed over traditional media forms. When the ban was lifted, it was made public through mass media.

It is also clear that over the five day period of imminent threat, the events in Boston attracted attention from a population of online observers drawn from a large geographical area. The scale and location of this audience leads to questions of whether emotive messages (or any other specific thematic area) are passed on by those closest to the threat, or by curious onlookers from across the globe. Future research is needed to investigate the geographical nature of retransmitted messages to address questions about which audiences are retweeting what kinds of content. Such insights will add to knowledge on the networked effects of warning via social media, as one channel for terse communication. It will also contribute knowledge about key nodes that facilitated retransmission of topic-specific message dissemination under conditions of threat.

Consistent with prior research, we also find that message style contributes to predicted message retransmission. Message style is important for increasing clarity and delivering unambiguous, consistent information. These are largely elements that increase understanding and reduce misinterpretation or confusion. In this case, we found that the use of ALL CAPS to signify the subject of the message was a strong predictor of retweets. ALL CAPS within a message may increase the visibility of specific content; the use of signifiers such as UPDATE or MEDIA ADVISORY may reduce ambiguity about the content of the message.

In addition, the inclusion of a weblink remains a barrier to message retransmission. In both cases studied thus far, one a natural hazard event and this, a terrorist event, we have found that a messages that include a URL are less likely to be retweeted among the online public. Twitter users appear to prefer brief messages that are easily transmitted across the network. Messages that include a link, and therefore require additional steps to acquire valuable information, may be perceived as more time consuming (to access and download the link) and resource-intensive (in terms of bandwidth required) in contrast with soundbites delivered in 140 characters or less. Our results indicate that terse messages containing a URL have a negative effect on message retransmission. Terse message retransmission appears to be a selective activity among message receivers. Perhaps those messages that are less complex, requiring few steps to gain the maximum amount of information, are the most relevant to Twitter users during periods of imminent threat. Alternately, messages that include URLs may simply lead users to switch their attention from Twitter to some other site, with the consequence that many never return to retweet the initial message. Future research is needed to investigate users’ motivations to pass along a message with or without a URL.

Finally, account features, most specifically Follower numbers, remains a strong predictor of retweet activity, suggesting that increasing connections among Followers is extremely valuable. While message design features remain key components to communicating specific content, message visibility via high Follower numbers is crucial for amplification across a broad spectrum of individuals.

## Conclusions

This study contributes to the growing body of work on allocation of attention and on message retransmission during hazard events. We find that, rather than any single effect dominating the process, retransmission of official Tweets during the Boston bombing response was jointly influenced by message content, style, and sender characteristics. This implies that simplistic models based e.g. only on network properties are unlikely to correctly predict which messages will be amplified in similar settings. On the other hand, relatively simple heuristics incorporating all three feature types may potentially perform well (as suggested by the large effect sizes observed in our fitted model).

Our research suggests that messages that include content on hazard impact and advisory information are likely to be highly salient with an online audience in an unfolding emergency. Furthermore, under conditions of terrorist threat, messages promoting community resiliency appear to have a highly positive effect on public retransmission. Importantly, messages that include a URL will decrease predicted rates of message retransmission. These findings suggest that there is a specific set of messages that can be preliminarily drafted to meet the standards and character limitations of terse messaging formats, prior to an event occurring. As we learn more about appropriate protective actions for terrorist attacks, very specific instructional messages can be created to prevent further casualties and to shore up resiliency to future attacks.

Terse messaging under conditions of imminent threat will require sophisticated planning on behalf of public communicators. As Sellnow and Sellnow state, [69] (p. 124), “the acute phase of a crisis leaves little time for traditional dialogue.” It also leaves little time for decision making about how to communicate key messages that will meet the public safety needs of those at risk and address the ongoing issues that arise in response to imminent threat, or time to grow one’s Follower numbers to reach more individuals. This research builds upon previous findings, demonstrating consistent patterns of message saliency among the public online while identifying key content areas specific to communicating during a terrorist event; our findings can help to shape message design strategies for crisis communications on terse messaging channels. As more agencies adopt Twitter and other terse messaging platforms such as Short Messaging Services for broadcast alerts in a disaster context, communication strategies must include attention to effective message elements that will appeal to a broad audience capable of amplifying messages through retransmission while simultaneously growing a broad Follower base.

## Supporting Information

S1 CSV Data table. Table containing coded tweet information and retweet counts.
(CSV)
